# Recent fall Eurasian cooling linked to North Pacific sea surface temperatures and a strengthening Siberian high

**DOI:** 10.1038/s41467-020-19014-2

**Published:** 2020-10-15

**Authors:** Baofu Li, Yupeng Li, Yaning Chen, Baohuan Zhang, Xun Shi

**Affiliations:** 1 School of Geography and Tourism, Qufu Normal University, 276826 Rizhao, Shandong Province China; 2 State Key Laboratory of Desert and Oasis Ecology, Xinjiang Institute of Ecology and Geography, Chinese Academy of Sciences, 830011 Urumqi, China; 3 Department of Geography, Dartmouth College, Hanover, NH 03755 USA

**Keywords:** Climate sciences, Atmospheric science, Atmospheric dynamics, Climate change

## Abstract

Winter Eurasian cooling after the mid-1990s has been verified by numerous studies, although in recent decades, the mid-latitudes of the Northern Hemisphere have been rapidly warming globally. Because the cooling is not uniform at different spatial and temporal scales, over time, this change may not truly reflect the nature of climate fluctuations. Here, by using three types of data (reanalysis, weather station, and remote sensing image data) to assess variations in Eurasian seasonal cooling, we examine the causes of these changes. During a 30-year climatology (1989–2018), we show that a significant (*P* < 0.05) abrupt change in the autumn Eurasian air temperature trend occurred in 2003. Our results suggest that from 2004–2018, the autumn Eurasian temperature reveals a significant cooling trend (*P* < 0.05). We demonstrate that the autumn cooling in Eurasia is likely influenced by the Pacific Decadal Oscillation (PDO) and Siberian high (SH). Since 2004, the strengthening of the PDO and SH explains approximately 54% and 18% of the autumn cooling in Eurasia, respectively. We also find that the cooling in autumn is stronger than that in winter.

## Abstract (web summary)

In the last years, an extensive winter cooling over central Eurasia has been discussed widely. Here, the authors show that from 2004–2018, the Eurasian cooling in autumn is stronger than that in winter, and that this autumn cooling is likely influenced by the Pacific Decadal Oscillation and the Siberian high.

## Introduction

Although global warming is an unquestionable fact^1,2^, numerous studies have found an unexpected phenomenon of extensive winter cooling over central Eurasia (CEU) in recent years^3–7^. Since the mid-1990s, the primary cooling trend in CEU occurs in winter (December–February)^8^, especially from 1995–2013^9–11^. As cooling trends on different temporal scales are inconsistent, these trends may not fully reflect the nature of climate variability over time^2^. Thus, it is important to diagnose the evolution of Eurasian cooling at different temporal scales.

In recent years, many studies^3–9^ have performed research on the physical mechanism of winter Eurasian cooling. However, no unified understanding has been reached^8,12^. Some studies believe that the decrease in sea ice is an important cause of winter cooling^6,9,11^. However, other studies oppose this theory^4,5^. Some studies have suggested that atmospheric circulation fluctuations are the main causes of winter cooling^5,13,14^. Thus, the explanations for the seasonal cooling phenomenon are complex and diverse. To achieve comprehensive climate system change, we must understand the scientific significance of this phenomenon.

This study presents evidence of autumn cooling in Eurasia. Our results show that the cooling in autumn is stronger than that in winter. We find that since 2004 a significant cooling trend has been attributed to the changes in North Pacific sea surface temperatures and a strengthening Siberian high. The results also show that the mechanisms responsible for the cooling trends in fall differ from winter.

## Results

### Autumn temperature change

**Fig. 1:**
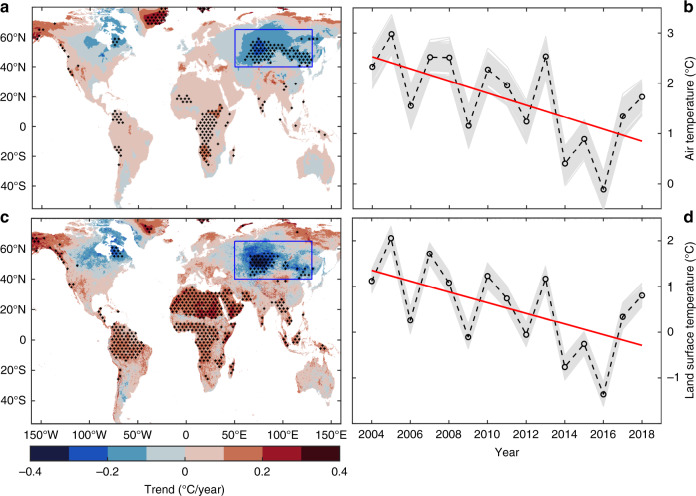
Temperature trends globally and in central Eurasia. **a** Autumn trends of ERA5-Land air temperatures from 2004 to 2018 in central Eurasia (CEU, 50–130° E, 40–65° N, enclosed by a blue rectangle). **b** Time series of ERA5-Land autumn air temperatures from 2004 to 2018. Each grey line (*n* = 500) is a time series of mean ERA5-Land air temperatures for a randomly selected ~1% (*n* = 2010) of the total grids for each time in CEU. **c** Autumn trends of MODIS land surface temperature from 2004 to 2018. **d** Time series of MODIS land surface autumn temperatures from 2004 to 2018. Each gray line (*n* = 500) is a time series of mean MODIS land surface temperatures for a randomly selected 1% (*n* = 8000) of the total grids in CEU. Black dots (**a**, **c**) represent significance at the *P* < 0.05 level. The black line is the average of the gray line data. The red line is the linear trend of the black line data (**b**, **d**).

Here, using climatological data from 1989–2018, we focus on how the trend of the land surface air temperature in CEU (50–130°E, 40–65°N, enclosed by the blue rectangle in Fig. 1) changed during the last 30 years. We use the latest atmospheric reanalysis data, ERA5-Land data, to analyze the 2-m air temperature changes in CEU from 1989–2018. The ERA5-Land data have higher spatial and temporal resolutions and more recent model and data assimilation systems than other reanalysis datasets^15,16^ (see the Methods for details). We use the Pettitt test to examine the abrupt changes in air temperature in the CEU. The analysis shows that a statistically significant abrupt change in the autumn average air temperature occurred in 2003 (*P* < 0.05, Supplementary Fig. 1a). Supplementary Fig. 1b also shows that the abrupt change in air temperature occurred in 2003, and covered the most grids. This accounted for 45.9% of the total grids, followed by 2001, which had a rate of 20.3%. Previous results^17^ have shown that abrupt changes in wind speed in Asia and Europe occurred in 2001 and 2003, respectively. There is a relationship between the wind speed and air temperature^17^, illustrating our results are credible. These results indicate an obvious difference in air temperature changes following these abrupt changes. Therefore, we focus on analyzing the temperature changes in CEU during 2004–2018.

We use the Mann–Kendall statistical test method to assess the significance of time series trends in temperature. In the time series, it shows that the autumn ERA5-Land mean air temperature in CEU displays a significant (*P* < 0.05) decreasing trend from 2004–2018 (Fig. 1b). To exclude the possibility that the trend is caused by strong temperature variations at only a few grids, we randomly sampled the analysis 500 times, which included ~1% (*n* = 2010) of the total grids. The air temperature displays obvious decreasing trends in every sample (gray lines in Fig. 1b).

Spatially, the results show that 99% of the area exhibits a cooling trend in autumn for the entire region of CEU from 2004 to 2018 (Fig. 1a). The Mann–Kendall test shows that the cooling in 32.3% of grids (*n* = 64,915, with an estimated area of 5,258,115 km^2^) is significant at the *P* < 0.05 level (black dots in Fig. 1a). To ensure the reliability of the results, we use the Monte Carlo method to quantify evaluations of field significance^18–20^. The results show that the cooling trends of air temperature reach field significance at the *P* < 0.01 level in CEU.

Furthermore, to avoid bias in the selected data, we use Moderate Resolution Imaging Spectroradiometer (MODIS) land surface temperature (LST) data to further verify the results. MODIS LST data have a high spatial resolution (5 × 5 km) and have been verified in many areas^21–23^. The mean LST in this region has a strong consistency with the mean ERA5-Land air temperature (Fig. 1d). Based on the LST data, we can also see a significant (*P* < 0.05) cooling trend in autumn. Clearly, the LST of 500 random samples 1% (*n* = 8000) of the total grids shows a downward trend (gray lines in Fig. 1d).

Spatially, 86.8% of the grids exhibit a cooling trend in autumn LST (Fig. 1c). The Mann–Kendall test shows that the cooling at 24.2% (*n* = 193,175, with an estimated area of 4,829,375 km^2^) of grids is significant at the *P* < 0.05 level (black dots in Fig. 1c). The estimates of field significance show that the cooling trends of LST in CEU reach significance at the *P* < 0.01 level.

**Fig. 2:**
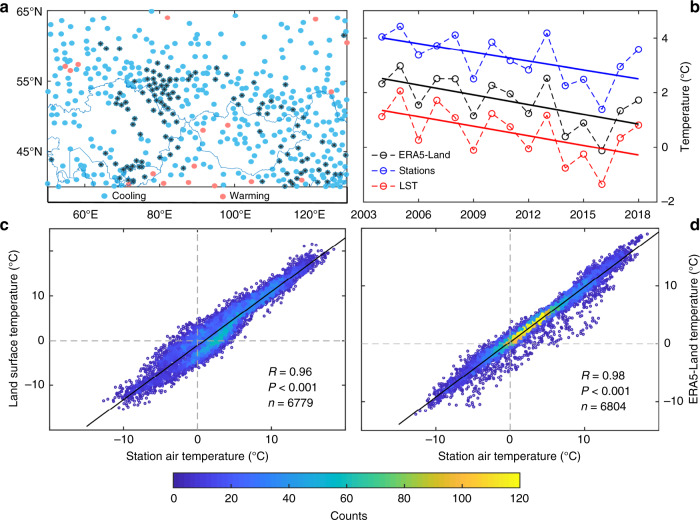
The autumn temperature trends and the relationship between different data. **a** The air temperature trends in autumn at 474 meteorological stations (each dot represents a meteorological station) in central Eurasia (CEU) from 2004–2018. Blue dots represent cooling, and pink dots represent warming. Black dots represent significance at the *P* < 0.05 level. **b** Comparison of average station temperature time series with land surface temperature (LST) and ERA5-Land air temperature. **c** Correlation between autumn air temperatures at 474 stations and their corresponding MODIS LST. **d** Correlation between autumn air temperatures at 474 stations and their corresponding ERA5-Land air temperatures. The counts of colours represent the number of samples in the same location.

Finally, we choose 474 weather stations (dots in Fig. 2a) from the Global Summary of Day (GSOD) database to further verify the reliability of the ERA5-Land and MODIS results. The quality of the weather station observation data is deemed dependable (Supplementary Fig. 2, see the Methods for details). In the time series, the autumn mean air temperature, at 474 stations, also displays a significant (*P* < 0.05) cooling trend (blue lines in Fig. 2b) from 2004–2018. Spatially, 96.4% of the station air temperature values exhibit a cooling trend in autumn (blue dots in Fig. 2a). The cooling trend at 24.9% stations reaches significance at the *P* < 0.05 level (black dots in Fig. 2a). Estimates of field significance also show that the results of the temperature decrease trend held for CEU (*P* < 0.01). These results once again confirm the autumn cooling in Eurasia.

Additionally, the correlation between the autumn air temperatures at 474 stations and their corresponding ERA5-Land air temperatures (correlation coefficient: *R* = 0.98) and LST (*R* = 0.96) reach significance at the *P* < 0.001 level (Fig. 2c,d), indicating that the ERA5-Land and MODIS data are highly reliable.

It should be noted that the potential data uncertainties may have some impacts on the study. For example, ERA-I-Land tends to underestimate albedo compared to observations, thus impacting surface temperatures^24^. The ERA5-Land soil-temperature is predicted to be colder than the observations in mid-low latitudes with an average bias of −0.08 °C^25^. The error rate of MODIS cloud detection at night is higher than that during the daytime^26^. There are few meteorological stations and they are unevenly distributed.

### Potential causes of autumn cooling

There is no consensus on the extent of central Eurasia, for example, CEU_M (60–120°E, 40–60°N)^4,9^ and CEU_S (80–120°E, 40–65°N)^27,28^. We choose the region where the cooling in autumn is relatively obvious as our study area (CEU, 50–130°E, 40–65°N, enclosed by a blue rectangle in Fig. 1a). Thus, the extents of these three regions are CEU, CEU_M, and CEU_S in descending order (Supplementary Fig. 3). To avoid different results caused by differences in the study area selections, we compare the causes of air temperature changes in the three regions.

**Fig. 3:**
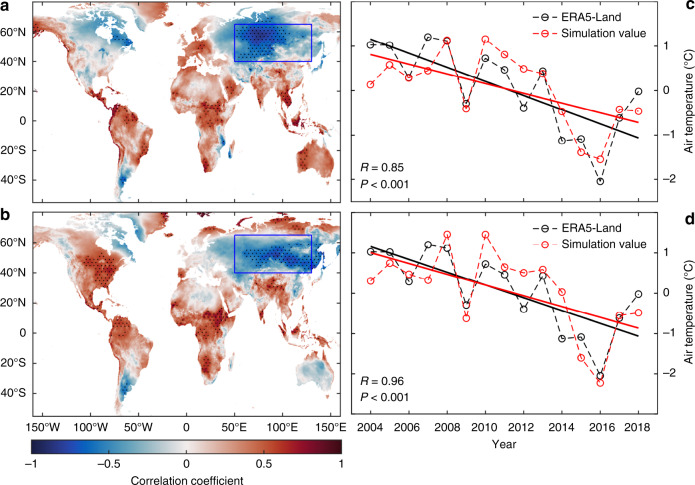
Spatial and temporal patterns of temperature and atmospheric circulations. **a** Spatial patterns of the correlation between the ERA5-Land air temperature and Pacific Decadal Oscillation (PDO) in autumn from 2004–2018. Black dots represent significance at the *P* < 0.05 level. **b** Spatial patterns of the correlation between the ERA5-Land air temperature and Siberian high (SH) in autumn from 2004–2018. **c** Temporal patterns of the ERA5-Land air temperature and temperature simulated by the binary linear regression equation based on the relationship between the air temperature and PDO and SH in autumn from 2004–2018. **d** Temporal patterns of the ERA5-Land air temperature and temperature simulated by the binary linear regression equation based on the relationship between the air temperature and the PDO and SH in autumn from 1989–2003. Temporal patterns of the ERA5-Land temperature, PDO and SH are analyzed based on the data after the global mean temperature (land and sea) forcing signal is removed (**c**, **d**).

Many studies^14,17,29,30^ have shown that the transport of heat by atmospheric circulations reduces the temperature heterogeneity at the Earth’s surface and this process also affects the regional climate. To determine which circulation has the greatest influence on the temperature in the three regions, we first analyze the correlation between the air temperature and 23 major atmospheric circulations (Supplementary Note 1) and 2 sea ice indexes (Supplementary Table 1a). The Pearson correlation coefficient values indicate that the autumn temperature in the three regions has had a strong and significant correlation with the Pacific Decadal Oscillation (PDO, *R* = −0.63 to −0.70, *P* < 0.01) and the Siberian High index (SHI, *R* = −0.60 to −0.69, *P* < 0.01) since 2004. For all the other tested atmospheric circulations, the correlations are much weaker and less significant. The spatial pattern of the autumn temperature in CEU is correlated with the PDO (Fig. 3a) and SHI (Fig. 3b). Clearly, there is a significant (*P* < 0.05) negative correlation between the PDO, SHI and air temperature in most areas of the CEU (black dots in Fig. 3a, b).

Simultaneously, we examine the correlation between the air temperature and circulation time series after the global (land and sea) warming trend is removed from the data (Supplementary Table 1b)^31^. The results show that the largest correlation coefficients are found between air temperature in the three regions (CEU, CEU_M, CEU_S) and PDO (*R* = −0.66 to −0.73, *P* < 0.01) and SHI (*R* = −0.64 to −0.72, *P* < 0.01), which increase slightly. This result indicates that PDO and SH have a clear influence on temperature.

To avoid overfitting, we further apply stepwise regression to identify the most significant factors to explain the changes in the air temperature in the three regions. The global warming trend is removed from all the data. The significance level we set for the regression model is 0.05. The PDO, SHI, and NAO data are input into the regression model, and their contribution rates to the temperature change of CEU_S are 43.8%, 19.5%, and 12.2%, respectively. Concurrently, only the PDO and SHI data are input into the stepwise regression model, and their contribution rates to the air temperature change in CEU (CEU_M) are 53.8% (51.6%) and 17.9% (25.0%), respectively. These results suggest that the PDO is the most significant explanation for autumn cooling in the three regions, followed by the SHI. Figure 3c shows the temporal patterns of the ERA5-Land air temperature and temperature simulated by the stepwise regression model based on the relationship between the air temperature in CEU and the PDO and SHI in autumn from 2004–2018. In general, the simulated air temperature is well synchronized with the ERA5-Land temperature from 2004–2018, reflecting a direct impact of the PDO and SHI on the temperature. A recent study^17^ shows that the most notable explanation for the increase in wind speed in Asia after 2001 is the strengthening of the PDO. This finding suggests that the PDO has an important influence on heat transfer in the study area.

Finally, we train the binary linear regression model using only the PDO, SHI, and temperature time series before the abrupt change in 2003 (1989–2003) and use this model to simulate the changes in air temperature from 2004–2018 based on the PDO and SHI data (Fig. 3d). We find that the simulated values could accurately reproduce the autumn temperature changes in the CEU. The correlation between the simulated temperature and the real value is highly significant (*R* = 0.96, *P* < 0.001). These results suggest that a predictive relationship exists between the ocean–atmosphere oscillations (e.g., the PDO and SHI) and the temperature in CEU.

## Discussion

Almost all previous studies^3–6,9–11^ have suggested that the cooling in CEU since the mid-1990s has mainly occurred during winter. However, we find that most areas of CEU in autumn have been cooling from 2004–2018. Supplementary Fig. 4 shows that 74% (*n* = 148,857, with an estimated area of 12,057,417 km^2^) and 88% (*n* = 177,160, with an estimated area of 14,349,960 km^2^) of the grids exhibits a warming trend in spring and summer, respectively. It is worth noting that 69% (*n* = 138,580, with an estimated area of 11,224,980 km^2^) of the grids also show a warming trend in winter. Notably, since 2004, the proportion of the cooling area in autumn has been much greater than that in winter, and the mean temperatures in spring and summer have been increasing. We thus suggest that the Eurasian cooling trend is no longer limited to winter, and we need to focus on the autumn cooling trend in CEU.

Although many studies have focused on winter cooling in CEU, there are still some disputes over the corresponding physical mechanism^4,8^, specifically regarding whether winter cooling is attributable to melting sea ice^5,32–37^. However, the theory that cooling is a result of melting sea ice is unlikely to support the autumn cooling observed during 2004–2018. Concurrently, many studies^4,5,10^ have focused on the effects of atmospheric circulations on CEU seasonal cooling, and specifically the effects of the Arctic Oscillation (AO)^12,13,38,39^ and North Atlantic Oscillation (NAO)^5^. The changes in the AO account for more than half of the surface air temperature variations in Eurasia from 1979–1997^40^. The AO, which is associated with the westerly winds, blocking frequency, Siberian high, and Rossby wave activities, is related to Eurasian temperatures, and this knowledge is generally accepted^12,40^. Therefore, the negative phase of the AO is associated with winter cooling in CEU^12,13,38–40^.

**Fig. 4:**
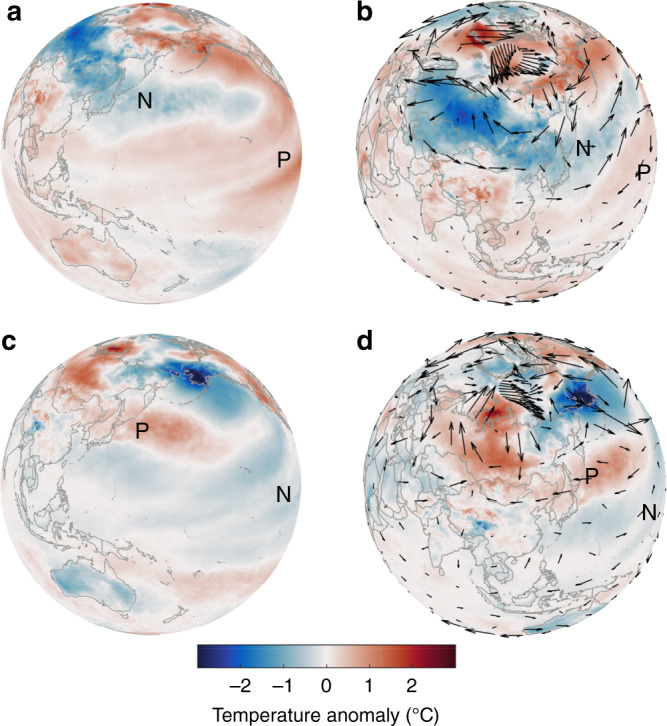
Mechanisms for the variations in autumn air temperature. **a** Spatial patterns of the positive phase of the Pacific Decadal Oscillation (PDO). **b** Spatial patterns of the wind field (black arrows) anomaly at 500 hPa and temperature anomaly (shading) with the PDO-positive phase. **c** Spatial patterns of the PDO-negative phase. **d** Spatial patterns of the wind field (black arrows) anomaly at 500 hPa and temperature anomaly (shading) with the PDO-negative phase. The letters ‘P’ and ‘N’ represent positive and negative phase temperatures, respectively.

However, we find that autumn cooling in the CEU is mainly due to the strengthening of the PDO and SHI. First, previous results^41,42^ have also shown that an out-of-phase relationship in the variability of the eddy temperature between Asia and the North Pacific is associated with the out-of-phase relationship in atmospheric heating. Second, many studies^43,44^ have shown that Arctic warming has an impact on the change in Eurasian temperatures. Simultaneously, some studies^45^ have suggested that the PDO is one of the major drivers of rapid Arctic warming in the early 20th century. Figure 4 also shows that the warm Arctic corresponds to the positive phase of the PDO. These results indirectly indicate that the PDO is an important influencing factor of temperature changes in Eurasia.

Some potential physical mechanisms that could clarify the effect of the PDO and Siberian high on CEU temperatures are given^29,46,47^. First, the positive PDO phases may cause the East Asian trough to strengthen and deepen (Fig. 4a, c). The growth and peak stages of the strong East Asian trough may lead to below-normal air temperatures over East Asia^48,49^. Second, Fig. 4b shows that the 500 hPa winds exhibit an easterly anomaly over the cold surface temperature anomalies, with westerlies to the south. The strengthening of the PDO generates a westerly surface wind component that increases the prevailing westerly jet at mid-latitudes^29,46^. The enhanced westerly jet further leads to an increase in cold northerly winds in Asia^50,51^ and eastern Europe (Fig. 4b, d). Finally, the Siberian high is a very cold dry anticyclone, which provides a cooling condition in the study area^27,52,53^, thus contributing to the areal-averaged temperature values.

In summary, we find that the autumn CEU temperature shows a significant (*P* < 0.05) downward trend during 2004–2018. We also suggest that the PDO and SHI are closely related to autumn cooling, and the driving mechanisms of cooling in Eurasia are somewhat different in autumn and winter. These results provide a comprehensive understanding of regional climate fluctuations.

In this study, we focus on some changes in Eurasian seasonal cooling and the effects of atmospheric circulations on these changes. We know that these changes in climate systems are the result of a combination of different factors. For example, there is a connection between snow cover and the atmosphere. Air temperature forcing causes variability in snow cover extent in the Northern Hemisphere^54,55^, while changes in snow cover can affect the phase and amplitude of atmospheric circulations (e.g., AO/NAO/SH)^56–59^. However, there are still many challenges in understanding snow-atmosphere coupling^55^. Thus, the complex physical mechanisms associated with seasonal cooling require further study.

## Methods

### Temperature datasets

A reanalysis dataset of 2-m air temperatures is obtained based on the ERA5-Land monthly averaged data from 1989–2018. ERA5-Land is a global atmospheric reanalysis product produced by the ECMWF (European Centre for Medium–Range Weather Forecasts)^60^. A series of improvements to the ERA5 climate reanalysis product have been added to the land component database. These improvements have increased the accuracy of the data for all types of land applications^16^. For example, ERA5-Land operates at an enhanced resolution (9 vs 31 km in ERA5). The trends of the reanalysis data combined with global observations are consistent with the general trends of other physical datasets. We can present an accurate description of the historical climate in ERA5-Land data from the past several decades. In addition, the global (land and sea) surface temperature in Fig. 4 is obtained from the monthly data of ERA5. ERA5 is a global atmospheric reanalysis product produced by the ECMWF with a spatial resolution of 0.25°^61^. We define spring as March–May, summer as June–August, autumn as September–November, and winter as December–February.

The autumn LST from 2004 to 2018 is calculated based on the monthly LST of the Surface Energy Balance Based Global Land Evapotranspiration database^23,62^. The monthly LST is produced from MODIS emissivity data (MOD11C3 V5 and MYD11C3 V5)^23,63,64^. MOD11C3 V5 and MYD11C3 V5 data have been verified under a range of representative conditions, with an average deviation of less than 1 K^65^. The monthly LST has a 0.05° grid size, without gaps, and covers the period from March 2000 to 2018; the data include monthly night-time and daytime LST values^23,66^. The monthly LST is calculated by averaging the daytime and night-time values of MOD11C3 and MYD11C3.

The autumn air temperatures at weather stations from 2004–2018 are calculated based on the GSOD database processed by the US National Climatic Data Center (ftp://ftp.ncdc.noaa.gov/pub/data/gsod). The database is obtained from the United States Air Force DATSAV3 surface dataset and the Federal Climate Complex Integrated Surface Hourly dataset. The analysis is based on data exchanged through the World Meteorological Organization (WMO) World Weather Watch Programme according to WMO Resolution 40 (Cg-XII)^17,67^. More than 400 algorithms are used for the quality control of temperature data from weather stations (see www.ncdc.noaa.gov/isd for details). The weather station data are available on a daily time step. We utilize the average monthly air temperature from stations that collect data for 15 days or more. Next, we calculate the autumn average temperature based on the monthly temperature for all stations (*n* = 642). Finally, one station with autumn temperature data missing for more than 5 years between 2004 and 2018 is removed. We select 474 stations to represent the station temperatures in the study area (for the distribution, see the dots in Fig. 2a). The quality of the autumn weather station data is shown in Supplementary Fig. 2. Seventy-seven percent of stations have no missing autumn air temperature data.

### Other datasets

The mean sea level pressure is used to calculate the Siberian high index^27^ from the monthly data of ERA5. The global (land and sea) 500 hPa wind field in Fig. 4 was obtained from ERA5. The autumn sea ice index from 1989 to 2018 is calculated based on the monthly sea ice extent/sea of the National Snow and Ice Data Center database^68^. Monthly sea ice extent/sea ice can effectively reveal changes in Arctic sea ice.

### Climate indices

Climate indices can describe the dynamics of ocean–atmospheric circulations^17^. We select 23 climate indices from 1989–2018 that reflect atmospheric and oceanic phenomena to analyze changes in atmospheric circulation with variations in the CEU temperature (Supplementary Note 1). These indices are widely used in climatological studies and represent the major atmospheric circulations in the Earth system.

### Analysis

The Pettitt test is applied to determine the abrupt changes in the autumn air temperature. We use the Mann–Kendall statistical method to assess the significance of the mean temperature trends in the study area. The Mann–Kendall statistical test has been commonly used to assess the significance of monotonic trends in climatic series^30^. The Monte Carlo method^18–20^ is used to estimate the field significance of the temperature cooling trend in the CEU.

To compare the temperature trends in the study periods, long-term temperature trends are obtained based on the slope of the linear regression line of the mean air temperature.

To check the reliability of the temperature data, we use the nearest neighbor algorithm to interpolate the ERA5-Land and MODIS data to obtain the temperature corresponding to 474 station locations. The correlation between autumn air temperatures at 474 stations and their corresponding ERA5-Land air temperature or MODIS land surface temperatures is calculated (Fig. 2c, d).

In studying the causes of air temperature change, we remove the global warming trend from all data. The global mean (land and sea) surface temperature is used as a proxy for external forcing signals^31^. This is based upon the regression of climate data onto the global mean surface temperature signal and the removal of the regressed counterpart from the data^69^. The global land temperature values come from ERA5-Land, and the global sea surface temperature data are from ERA5.

Considering the temperature and each of the climate indices in this study, we calculate Pearson’s correlation coefficient (*R*) to determine the corresponding correlation (Supplementary Table 1). This statistic is also calculated for the correlation between ERA5-Land, MODIS LST, and weather station temperature values.

We utilize a forward stepwise regression algorithm^17^ to determine the main climate indices that had the largest effect on the temperature changes in autumn from 2004–2018. To determine which predictors should be included, we compare the explanatory powers of large and small models. The values of the *P* and *F* statistics are calculated in each step to assess models that may include a predictor not already supported by the model. For the regression model, we set the significance level at *P* < 0.05. If included in the model, the null hypothesis will have a zero-coefficient predictor. The predictor will be added to the model if there is enough evidence to reject the null hypothesis (at a given significance level). The greater the explanatory power of the predictor is, the earlier the predictor enters the model.

A binary linear regression method is used to establish the relationship between the autumn temperature and the PDO and SHI from 1989–2003; this model is used to simulate the autumn temperature change based on the PDO and SHI time series from 2004 to 2018 (Fig. 3d).

## Supplementary information

Supplementary Information

## Data Availability

The ERA5-Land and ERA5 data are downloaded from the Climate Data Store (https://cds.climate.copernicus.eu/). The air temperatures of 474 stations are from the Global Surface Summary of the Day database (GSOD, ftp://ftp.ncdc.noaa.gov/pub/data/gsod). The time series of MODIS LST are obtained from the National Tibetan Plateau Data Center (http://data.tpdc.ac.cn). The MOD11C3 and MYD11C3 data are from the NASA MODIS Web (https://modis.gsfc.nasa.gov/). The monthly PDO data are downloaded from the China National Climate Center (https://cmdp.ncc-cma.net/cn/index.htm). The other monthly climate indices are obtained from the National Oceanic and Atmospheric Administration (https://www.esrl.noaa.gov/psd/data/climateindices/list/). The monthly sea ice index data are obtained from the National Snow and Ice Data Center (NSIDC; https://nsidc.org/). All datasets are also available upon request from B.L.

## Code availability

All code to reproduce the results of this study are available on reasonable request from the corresponding author.
