# Supplementary material for: Recent fall Eurasian cooling linked to North Pacific sea surface temperatures and a strengthening Siberian high
Source: Nat Commun. 2020 Oct 15;11:5202. doi: 10.1038/s41467-020-19014-2 (PMC7567785; doi:10.1038/s41467-020-19014-2)
Supplement: Supplementary file 1 — Supplementary Information [file 41467_2020_19014_MOESM1_ESM.pdf]

Supplementary Information for

Recent fall Eurasian cooling linked to North Pacific sea surface temperatures and a strengthening Siberian high

Baofu Li[1*], Yupeng Li[2], Yaning Chen[2*], Baohuan Zhang[1], Xun Shi[3]

[1] School of Geography and Tourism, Qufu Normal University, Rizhao, Shandong Province 276826, China.

[2] State Key Laboratory of Desert and Oasis Ecology, Xinjiang Institute of Ecology and Geography, Chinese Academy of Sciences, Urumqi 830011, China.

[3] Department of Geography, Dartmouth College, Hanover, NH 03755, USA.

*Correspondence to: libf@qfnu.edu.cn (B. Li); chenyn@ms.xjb.ac.cn (Y. Chen)

Supplementary Figure 1 Abrupt changes for autumn air temperature in central Eurasia. (**a**) Abrupt change test for mean autumn air temperature in ERA5-Land for central Eurasia (CEU) in recent 30 years climatology (1989-2018) by use of Pettitt test. (**b**) Proportional distribution of the estimated abrupt changes derived from every grid result in the study area (*n* = 200,739) in recent 30 years climatology (1989-2018) by use of Pettitt test.

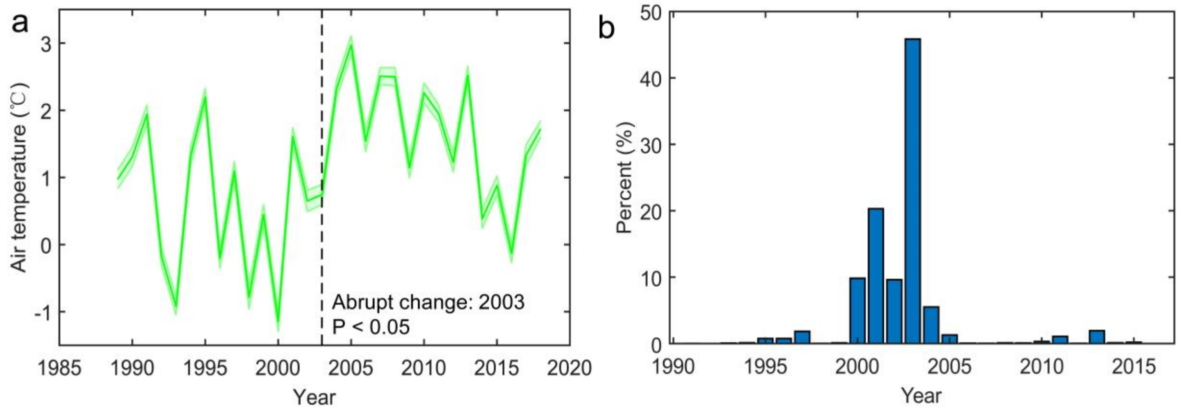

Supplementary Figure 2 Quality of weather stations data from 2004 to 2018 for autumn. (**a**) Proportional distribution of missing values in autumn air temperature at 474 weather stations during 2004-2018. (**b**) Distribution of autumn air temperature time series at 474 stations and orange lines represent missing values.

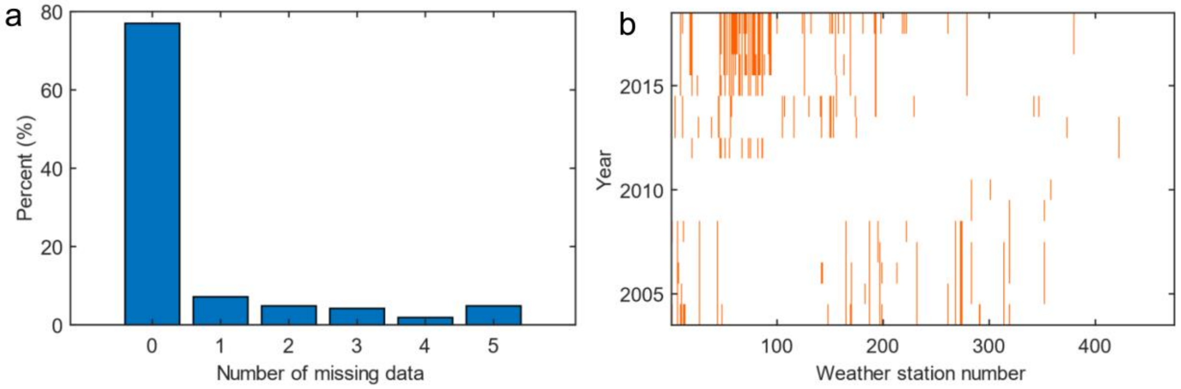

Supplementary Figure 3 Different borders in central Eurasia. CEU (central Eurasia, 50-130°E, 40-65°N), enclosed by the blue rectangle. CEU_M (medium central Eurasia, 60-120°E, 40-60°N), enclosed by the red rectangle. CEU_S (small central Eurasia, 80-120°E, 40-65°N), enclosed by the black rectangle. The trends of ERA5-Land air temperatures in autumn from 2004 to 2018 (shading). There is no consensus on the extent of central Eurasia. Some studies use relatively small CEU (CEU_S, 80-120°E, 40-65°N)[1,2], some use medium CEU (CEU_M, 60-120°E, 40-60°N)[3,4], while we choose the region where the cooling in autumn is relatively obvious as our study area (CEU, 50-130°E, 40-65°N). To avoid different results caused by differences in the study area selections, we compare the causes of air temperature changes in the three regions.

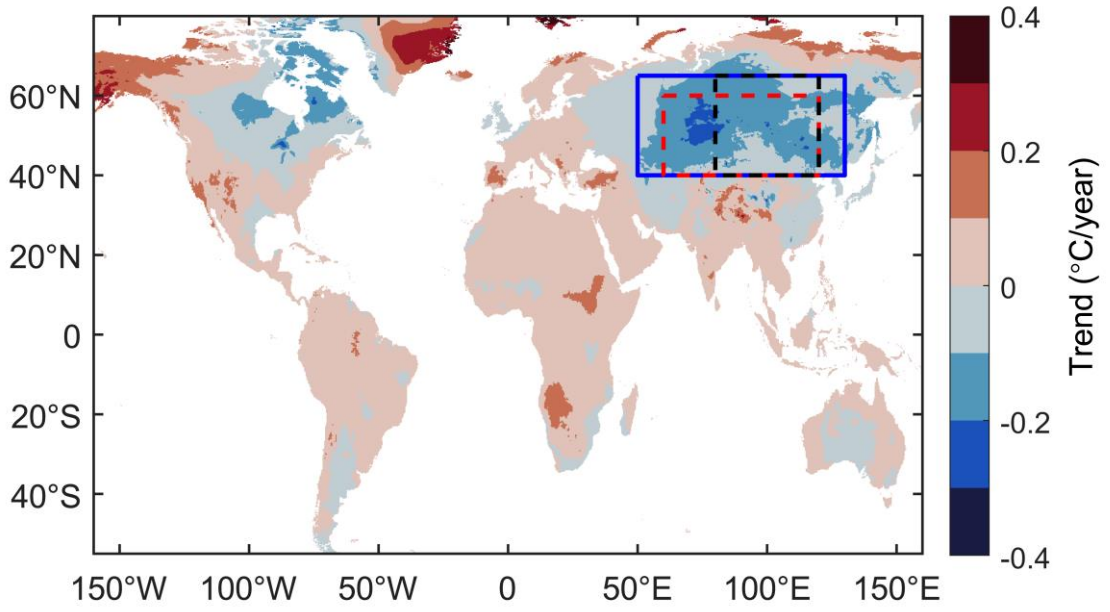

Supplementary Figure 4 The trends of the global and central Eurasia air temperature. The spatial pattern of air temperature in spring (**a**), summer (**c**), and winter (**e**) from 2004 to 2018. The time series of ERA5-Land air temperature in spring (**b**), summer (**d**), and winter (**f**) from 2004 to 2018. Each grey line ($n = 500$) is a time series of mean ERA5-Land air temperatures for a randomly selected approximately 1% ($n = 2010$) of the total grids each time in central Eurasia (CEU, 50–130° E, 40–65° N, enclosed by a blue rectangle). The change in the spatial distribution of temperature trends in winter is similar with what was shown in Supplementary Figure 2 of the previous study[5]. The gray band is composed of 500 gray lines.

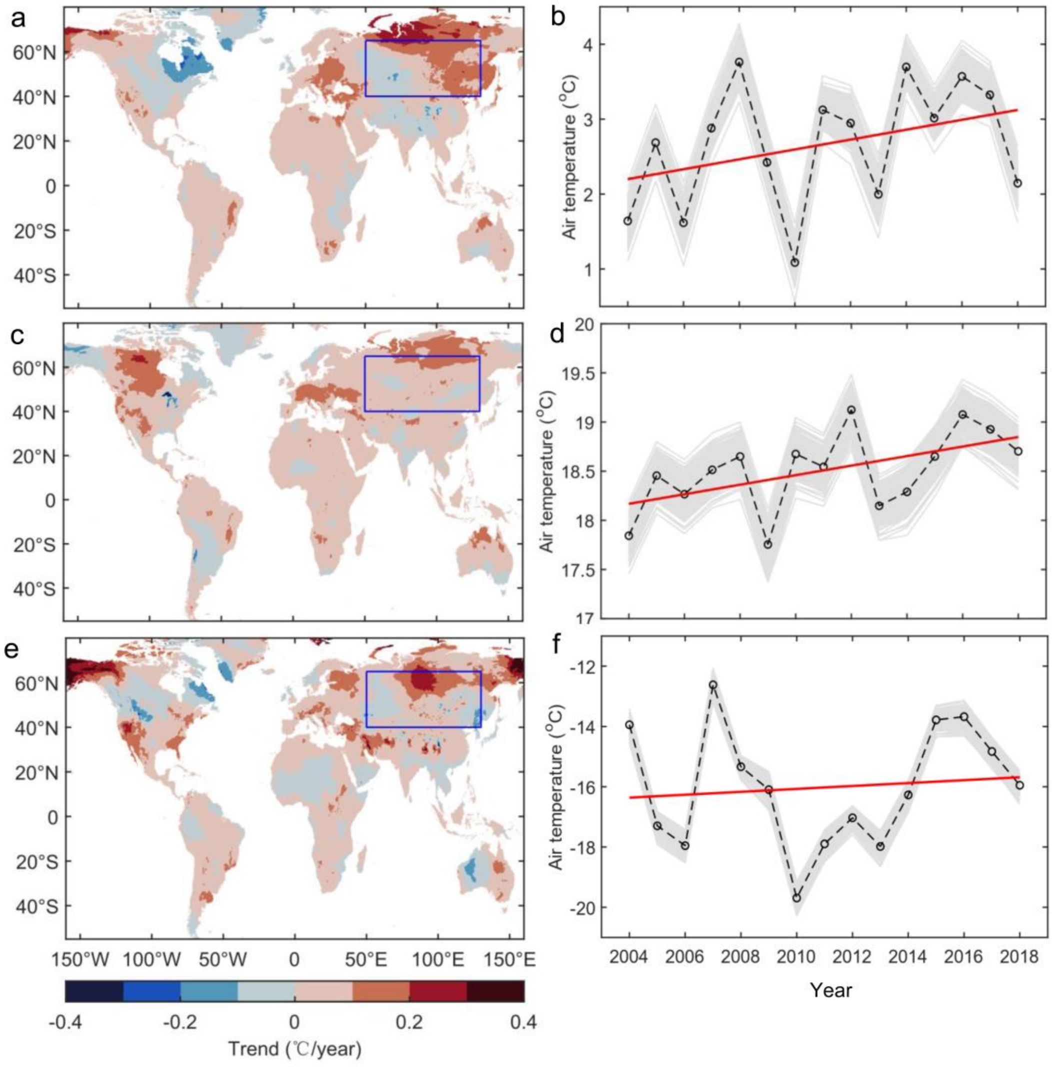

Supplementary Table 1 Correlation between autumn air temperature and atmospheric circulations. (**a**) Correlation coefficient between air temperature and atmospheric circulations (see the Supplementary Note 1 for details) during 2004-2018. (**b**) Correlation coefficient between air temperature and circulations after the global mean temperature (land and sea) forcing signal was removed. * significant at $P < 0.05$, ** significant at $P < 0.01$, *** significant at $P < 0.001$. CEU (central Eurasia): 50-130°E, 40-65°N. CEU_M (medium central Eurasia): 60-120°E, 40-60°N. CEU_S (small central Eurasia): 80-120°E, 40-65°N.

	a CEU	a CEU_M	a CEU_S	b CEU	b CEU_M	b CEU_S
PDO	-0.70**	-0.69**	-0.63**	-0.73***	-0.72**	-0.66**
SOI	0.48	0.47	0.44	0.47	0.46	0.43
Niño12	-0.58*	-0.52*	-0.47	-0.53*	-0.48	-0.42
TPI2	-0.46	-0.43	-0.46	-0.44	-0.41	-0.44
AO	0.53*	0.49*	0.56*	0.50*	0.47	0.53*
NP	0.39	0.42	0.26	0.29	0.33	0.18
Niño3.4	-0.34	-0.35	-0.36	-0.31	-0.31	-0.32
Niño4	-0.36	-0.38	-0.40	-0.32	-0.33	-0.34
WHWP	-0.61**	-0.58*	-0.61**	-0.53*	-0.46	-0.49*
TAN	-0.13	-0.01	-0.23	0.08	0.21	0.01
PNA	-0.05	-0.12	0.07	0.02	-0.03	0.14
EPNP	-0.15	-0.05	-0.14	-0.20	-0.13	-0.20
WP	0.45	0.49*	0.55*	0.36	0.39	0.44
NAO	-0.10	-0.24	0.04	-0.12	-0.23	0.02
TSA	-0.22	-0.24	-0.21	-0.09	-0.10	-0.08
ONI	-0.35	-0.36	-0.37	-0.32	-0.32	-0.33
TNI	-0.09	-0.01	0.07	-0.12	-0.06	0.02
AAO	0.11	0.11	0.14	0.09	0.09	0.12
AMO	-0.34	-0.26	-0.40	-0.19	-0.06	-0.20
NTA	-0.14	-0.03	-0.27	0.04	0.16	-0.04
MEI V2	-0.40	-0.40	-0.40	-0.41	-0.41	-0.41
Niño3	-0.38	-0.37	-0.36	-0.34	-0.32	-0.31
SHI	-0.60*	-0.69**	-0.61**	-0.65**	-0.72**	-0.64**
SIE	0.42	0.49*	0.45	0.14	0.15	0.12
SIA	0.45	0.51*	0.46	0.20	0.21	0.17

* P < 0.05
** P < 0.01
*** P < 0.001

Correlation coefficient: -1 0 1

Supplementary Note 1

PDO (Pacific Decadal Oscillation) is the leading PC of monthly SST anomalies in the North Pacific Ocean. SOI (Southern Oscillation Index) is a standardized index based on the observed sea level pressure differences between Tahiti and Darwin, Australia. Niño12 (Extreme Eastern Tropical Pacific SST) is an indicator of far eastern tropical Pacific El Niño conditions, off the coasts of Peru and Chile. It is calculated with SSTs in the box 90°W-80°W, 10°S-0. TPI2 (Tripole Index for the Interdecadal Pacific Oscillation) is based on the difference between the SSTA averaged over the central equatorial Pacific and the average of the SSTA in the Northwest and Southwest Pacific. Monthly AO (Arctic Oscillation) index is constructed by projecting the monthly mean 1000-hPa height anomalies onto the leading EOF mode. NP (North Pacific pattern) is the area-weighted sea level pressure over the region 30N-65N, 160E-140W. Niño 3.4 (East Central Tropical Pacific SST) is calculated with SSTs in the box 170°W-120°W, 5°S-5°N. Niño 4 (Central Tropical Pacific SST) is calculated with SSTs in the box 160°E-150°W, 5°S-5°N. WHWP (Western Hemisphere warm pool) is the monthly anomaly of the ocean surface area warmer than 28.5 °C in the Atlantic and eastern North Pacific. TAN (Tropical Northern Atlantic Index) is calculated with SSTs in the box 55°W-15°W, 5°N – 25°N. PNA (Pacific North American Index) is one of the most prominent modes of low-frequency variability in the Northern Hemisphere extratropics. EPNP (East Pacific/North Pacific) is a Spring-Summer-Fall pattern with three main anomaly centers. The positive phase of this pattern features positive height anomalies located over Alaska/ Western Canada, and negative anomalies over the central North Pacific and eastern North America. WP (West Pacific Index) is a primary mode of low-frequency variability over the North Pacific in all months. NAO (North Atlantic Oscillation) consists of a north-south dipole of anomalies, with one center located over Greenland and the other center of opposite sign spanning the central latitudes of the North Atlantic between 35°N and 40°N. TSA (Tropical Southern Atlantic Index) an indicator of the surface temperatures in the Gulf of Guinea, the eastern tropical South Atlantic Ocean. It is calculated with SSTs in the box 30°W-10°E, 20°S-0. ONI (Oceanic Niño Index) is the three months running mean of ERSST.v5 SST anomalies in the Niño 3.4 region (5°N-5°S, 120°W-170°W). TNI (Trans-Niño Index) is the standardized Niño 12 minus the Niño 4 with 5 months running mean applied which is then standardized using the 1950-1979 period. AAO (Antarctic Oscillation) is constructed by projecting the daily (00Z) 700mb height anomalies poleward of 20°S onto the loading pattern of the AAO. AMO (Atlantic Multidecadal Oscillation) is newly computed from a new dataset. The data is calculated from the Kalplan SST. NTA (North Tropical Atlantic SST Index): The timeseries of SST anomalies averaged over 60°W to 20°W, 6°N to 18°N and 20°W to 10°W, 6°N to 10°N. MEI V2 (Multivariate ENSO Index): Time series is bimonthly so the Jan value represents the Dec-Jan value and is centered between the months. Niño 3 (Eastern Tropical Pacific SST) is calculated with SSTs in the box 150°W-90°W, 5°S-5°N. SHI (Siberian high index) is defined as the mean sea level pressure averaged over the region (80-120°E, 40-65°N). SIE (sea ice extent) is the average sea ice extent in Arctic. SIA (sea ice area) is the average sea ice area in Arctic.

Supplementary References

1. Panagiotopoulos F, Shahgedanova M, Hannachi A, Stephenson DB. Observed trends and teleconnections of the Siberian high: A recently declining center of action. *Journal of Climate* **18**, 1411-1422 (2005).
2. Zhao S, et al. Impact of climate change on Siberian High and wintertime air pollution in China in past two decades. *Earth's Future* **6**, 118-133 (2018).
3. McCusker KE, Fyfe JC, Sigmond M. Twenty-five winters of unexpected Eurasian cooling unlikely due to Arctic sea-ice loss. *Nature Geoscience* **9**, 838-842 (2016).
4. Mori M, Kosaka Y, Watanabe M, Nakamura H, Kimoto M. A reconciled estimate of the influence of Arctic sea-ice loss on recent Eurasian cooling. *Nature Climate Change* **9**, 123-129 (2019).
5. Cohen J, Zhang X, Francis J, Jung T, Kwok R, Overland J, et al. Divergent consensuses on Arctic amplification influence on midlatitude severe winter weather. *Nature Climate Change,* **10**(1), 20-29 (2020).
